# DNA indels in coding regions reveal selective constraints on protein evolution in the human lineage

**DOI:** 10.1186/1471-2148-7-191

**Published:** 2007-10-12

**Authors:** Nicole de la Chaux, Philipp W Messer, Peter F Arndt

**Affiliations:** 1 Department for Computational Molecular Biology, Max-Planck-Institute for Molecular Genetics, Ihnestr. 63-73, 14195 Berlin, Germany; 2 Department for Biochemistry, University of Zurich, Winterthurerstrasse 190, 8057 Zürich, Switzerland

## Abstract

**Background:**

Insertions and deletions of DNA segments (indels) are together with substitutions the major mutational processes that generate genetic variation. Here we focus on recent DNA insertions and deletions in protein coding regions of the human genome to investigate selective constraints on indels in protein evolution.

**Results:**

Frequencies of inserted and deleted amino acids differ from background amino acid frequencies in the human proteome. Small amino acids are overrepresented, while hydrophobic, aliphatic and aromatic amino acids are strongly suppressed. Indels are found to be preferentially located in protein regions that do not form important structural domains. Amino acid insertion and deletion rates in genes associated with elementary biochemical reactions (e. g. catalytic activity, ligase activity, electron transport, or catabolic process) are lower compared to those in other genes and are therefore subject to stronger purifying selection.

**Conclusion:**

Our analysis indicates that indels in human protein coding regions are subject to distinct levels of selective pressure with regard to their structural impact on the amino acid sequence, as well as to general properties of the genes they are located in. These findings confirm that many commonly accepted characteristics of selective constraints for substitutions are also valid for amino acid insertions and deletions.

## Background

Molecular evolution is governed by the interplay of mutational processes which constantly give rise to the emergence of mutant alleles, and selective forces that influence the dynamics of mutants within the population, either leading to their fixation or loss. The decisive factor determining the probability of fixation of a new allele is its relative fitness compared to the wild type. If fitness differences are weak, the dynamics of a mutant within the population is essentially determined by genetic drift, reflecting stochastic fluctuations that result from a finite population size. In this regime of so called neutral evolution, the rate of fixation of new mutants in the population resembles the rate at which mutants are generated in individuals. On the other hand, if fitness differences between mutant and wild type are sufficiently large, stochastic fluctuations are overruled by deterministic selective forces which can lead to accelerated fixation of a beneficial mutant (positive selection), or its rapid removal as a consequence of strong selective constraints (purifying selection). These considerations have been put on a quantitative basis in the famous Kimura-Ohta theory of population genetics for finite populations evolving by stochastic fluctuations and selection [2].

As an immediate consequence of this theory, reduction of mutational rates in particular genomic regions compared to presumably neutrally evolving regions are indicative for selective constraints associated with the particular mutational processes. In our analysis, we use this approach to investigate selective forces on a specific class of mutational processes, insertions and deletions of short DNA segments in protein coding regions of the human genome.

Together with nucleotide substitutions and genome rearrangements, DNA insertions and deletions (indels) are the major mutational mechanisms to cause genetic variation. Comparative studies between human and chimp revealed that indels between both species cover approximately 3–5% of the two genomes, and therefore clearly outnumber the ~1.23% divergence resulting from single nucleotide substitutions [3-6]. Indels in human protein coding regions that occurred since the human-chimp split were measured to be highly suppressed compared to intergenic and intronic indels [7,8]. This finding reflects strong purifying selection in protein coding regions throughout recent human evolution. Coding indels in the human lineage should therefore provide a promising class of mutational processes to study the characteristics of selective constraints on protein coding regions in more detail.

We identified recent coding indels in the human lineage from whole-genome multiple alignments of human, chimp and rhesus. Insertions were explicitly distinguished from deletions using rhesus as an out-group species. All identified events were subjected to thorough quality filtering. The resulting set of reliable insertion and deletion events was analyzed under several aspects that reflect commonly regarded manifestations of selective constraints on protein evolution. Among them are variations in insertion or deletion rates between different amino acids, preferential occurrence of indels in specific secondary structure regions of proteins, and higher or lower rates of indels in genes associated with particular molecular functions, biological processes, or cellular components.

Our analysis indicates that indels in human protein coding regions are indeed subject to distinct levels of selective pressure with regard to their structural impact on the amino acid sequence, as well as to general properties of the genes they are located in. The results extend several known characteristics of selective constraints for amino acid substitutions [9,10] and indels in other species [11] to coding indels in the human lineage, and substantiate these findings by quantitative data.

## Results and discussion

### Insertion and deletion events

We investigated multiple alignments of the human, chimpanzee, and rhesus genomes to identify indels in the human branch since its split from the common ancestor with chimp. Using rhesus as an outgroup, indels were separated into insertions and deletions in the human branch by means of maximum parsimony [12]. To reduce the number of false-positive indels due to alignment or sequencing errors we applied rigid quality filtering on indels and their flanking regions (see methods). This way we identified a set of 188,379 insertions and 329,433 deletions in a total of 2747,5 Mbp (85%) of the human genome, which are covered by the multiple alignments.

In this set, 724 indels were detected to be located within protein coding sequence segments according to the Ensembl (version 41) annotation of the human genome [13]. Coding indels hence account for only 0.14% of all indels in our set. Comparison of this fraction with the density of protein coding segments, which is about 1.2% for the human genome [14], indicates that indels in coding regions are highly suppressed relative to those in the genomic background. This can be expected since coding indels will always change the amino acid sequence of the translated protein (in contrast to nucleotide substitutions, which can be synonymous). The effects of indels on the protein sequence can range from insertions or deletions of amino acids if indel lengths are multiples of 3 bp (non-frameshifting indels), up to complete non-functionalisation of the protein in case of frameshifting indels. Mutants carrying frameshifting indels are consequently more likely to be removed from the population by purifying selection than those with frameshifting indels [11]. Indeed, it has been found that the rates of frameshifting indels in protein coding regions are only about 5% of those in the genomic background, while non-frameshifting indels still occur at about 50% of their background rates [8].

Despite the approximately 10 times higher suppression of frameshifting indels compared to non-frameshifting indels, we still find 324 events (44.7%) in our set to be frameshifting. This is due to the fact that length distributions of insertions and deletions rapidly decay with increasing indel lengths [8]. For instance, on a genome-wide average 1 bp indels occur approximately 10 times more often than 3 bp indels. The number of 324 identified frameshifting indels is still unexpectedly high concerning their presumably profound impact on the translated protein sequence. One possible scenario could be that there is only a small number of wrongly predicted Ensemble genes that give rise to many frameshifting indels. Yet, this is not supported by our data; there is no gene containing more than two indels of our set, and only 9 (13) genes have two non-frameshifting (frameshifting) indels. Another likely origin of frameshifting indels could be falsely annotated coding regions. To further investigate this possibility, we checked the fraction of indels that are located in experimentally validated RefSeq peptides [15]. While 259 of the 400 non-frameshifting indels (65%) occured in exons of Ensembl transcripts that could be mapped to RefSeq peptides with at least 99% target and query identity, it was possible for only 108 of the 324 frameshifting indels (33%). This disproportionality is indeed indicative for a significant fraction of frameshifting indels being located in erroneously predicted Ensembl exons, but still a substantial number of events cannot be explained this way. So far, we are not able to rate what fraction of the remaining frameshifting events is biologically meaningful, and what are the contributions of alignment, sequencing, and other sources of error. In principle, it is also possible that a frameshift caused by one indel can be compensated by a second frameshifting indel. If both events occur within a close distance, changes in the amino acid sequence can be minimized.

**Figure 1:**
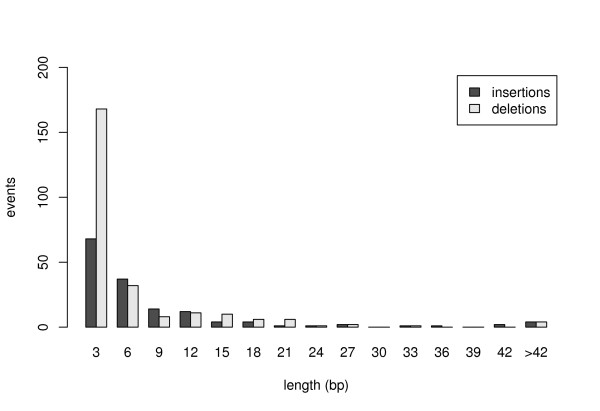
**Length distribution of non-frameshifting indels**. Length distributions of coding insertions and deletions decay rapidly with increasing indel length. The longest insertion in our set is a 405 bp long segment, the largest deletion covers 168 bp.

In contrast to frameshifting indels, which are generally "global" events causing changes on a protein scale, our analysis focuses on the contribution of indels to protein evolution on a "local" scale, and we therefore restricted our set to the 151 insertions and 249 deletions which are non-frameshifting. Their length distribution is shown in Figure 1; it is strongly peaked at 3 bp and rapidly decays for larger indel lengths. A table containing chromosomal position, length, and inserted/deleted sequence of all identified non-frameshifting indels in coding regions is provided online [1].

### Conservative and non-conservative indel events

**Figure 2:**
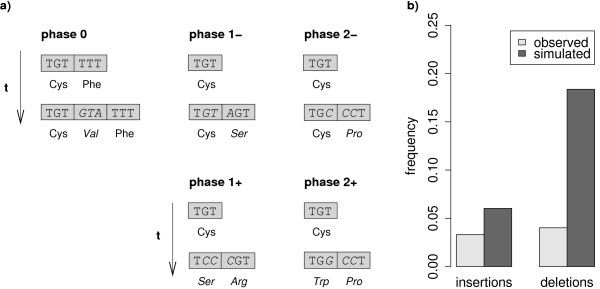
**Conservative and non-conservative indel events**. a) Examples of 3 bp insertions in a protein coding region. An insertion can either occur between two codons (phase 0), between the first and second nucleotide of a codon (phase 1), or between the second and third nucleotide (phase 2). Phase 1 and 2 insertions can thereby be divided into conservative events, which only insert a new amino acid without changing the translated amino acid of the ancestral codon (phase 1- and 2-), or non-conservative events that additionally change it (phase 1+, 2+). Insertions in phase 0 are always conservative. In a similar manner deletions can be partitioned into the 5 different categories (reversing time arrows in the figure yields the corresponding examples). Notice that the indel in phase 1-could have also been assigned as a phase 0 or phase 2 indel depending on where the alignment algorithm prefers to place the gap (all three gap placements have equal numbers of matches and gaps and therefore equal alignment scores). b) Measured frequencies of non-conservative insertion and deletion events in observed data and simulations.

Insertions and deletions can occur between two codons (in phase 0), or after the first or second nucleotide of a codon (in phase 1 and 2, respectively). Often, the exact phase of an indel cannot be reconstructed unambiguously on the sole basis of a multiple alignment. For instance, if an indel has occurred in a local repeat structure, the alignment algorithm has multiple possibilities to place the gap without changing the overall score of the alignment (see Figure 2a).

On the level of the translated amino acid sequence the different phases are not entirely equivalent: Non-frameshifting indels in phase 0 always introduce or delete complete codons of the protein coding sequence without affecting adjacent amino acids (conservative events). Indels in phase 1 or 2 can in addition to the inserted/deleted amino acids also change an adjacent amino acid (non-conservative events). Notice that phase 1 and 2 indels can also be conservative depending on the nature of the inserted or deleted sequence. In contrast to its exact phase, the conservative or non-conservative nature of an indel can unambiguously be determined from the multiple alignment. The classification into non-conservative and conservative events partitions indels into events causing an additional amino acid substitutions and those without. It is therefore a reasonable classification of indels whenever one is interested in their actual effect on the protein sequence.

In our set, we find non-conservative insertions and deletions to be strongly suppressed; they make up only 3% (5 events) of all insertions and 4% (10 events) of all deletions (Figure 2b). Indels in protein coding regions are hence predominantly of conservative nature, i. e. they occur in a way that minimizes the number of changed amino acids.

There are two hypothesis capable of explaining the strong bias towards conservative indel events: The first is a mechanistic explanation based on the observation that the majority of DNA insertions on short length scales are actually tandem duplications of adjacent sequence segments and that deletions also occur frequently in preexisting tandem duplicates [8,16]. These signatures are also found among the insertions and deletions in our set: 134 of 151 insertions (89%) are tandem duplications, 149 of 249 deletions (60%) removed one copy of a preexisting tandemly repeated motif. Non-frameshifting tandem duplication insertions and deletions of a repeated motif are always conservative events, irrespective of the phase they occur in. The measured overrepresentation of conservative indel events could hence be solely caused by the characteristics of the underlying molecular processes that generate indels.

The second hypothesis presumes that in addition to the previous "selectively neutral" explanation at least part of the observed strong bias may reflect a higher amount of purifying selection associated with non-conservative indels due to their larger number of effective changes in the protein sequence compared with conservative events.

To test whether the duplication mechanism can fully suffice to explain the observed underrepresentation of non-conservative indels we conducted a simulation study. Indels were thereby randomly placed in a test set of protein coding regions of the human genome. For each event the length of the simulated indel was drawn from the observed distribution of indel lengths in coding regions (Figure 1). As the crucial feature of our simulation we further assured that indels were generated with realistic duplication characteristics as observed on the genome-wide scale (see methods).

The measured frequencies of non-conservative events in our simulation are shown in Figure 2b. In comparison to the original set we obtained substantially higher frequencies of non-conservative events in the simulation set (insertions: 6%, deletions: 18%). For deletions, we can clearly reject the hypothesis that the small frequency of non-conservative events in our observed set is simply a result of the preferential deletion of copies within preexisting duplicates (*p *< 10^-9^, Fishers Exact Test). The corresponding statement is less significant for insertions (*p *< 0.3) due to the small number of predicted non-conservative events in our set (6%, amounting to 9 events in our set of 151 insertions). We actually observed 5 events. We conclude from this analysis that non-conservative indels are highly suppressed in protein coding regions. This can for a large part be explained by inherent duplication features of indels. However, non-conservative deletions are in addition subject to a significantly larger amount of purifying selection compared to non-conservative events, and a similar statement is likely to be true also for insertions.

### Inserted and deleted amino acids

**Figure 3:**
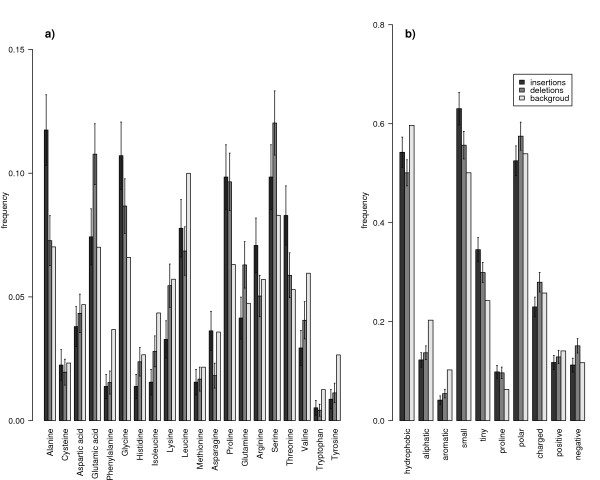
**Frequencies of inserted and deleted amino acids**. a) Frequency distribution of inserted/deleted amino acids resulting from coding indels in our set compared to the background amino acid frequencies in all human proteins. b) Frequencies of inserted/deleted amino acids grouped according to 10 different physio-chemical categories. Notice that amino acids can be assigned to more than one category. Error bars in a) and b) are standard deviations calculated by $\Delta {ƒ}_{i}=\sqrt{N_{i}}/\sum_{j}N_{j}$, where *N*_*i *_is the total number of inserted/deleted amino acids *i*, or amino acids in category *i*, respectively.

To investigate whether indels in protein coding regions preferentially induce insertions or deletions of specific amino acids, we counted the distributions of inserted and deleted amino acids in our set (see methods). These distributions were compared to the overall abundance of amino acids in proteins of the human genome. As shown in Figure 3a, both distributions are significantly different from the background abundance (*p *< 10^-14 ^for insertions, *p *< 10^-10 ^for deletions, Chi Square Test).

In particular, we found that glycine (*p *< 0.06, all p-values are corrected for multiple testing) and alanine (*p *< 0.02) were inserted more often than expected under the assumption that insertion frequencies of different amino acids follow the average distribution of amino acid frequencies in all coding regions of the human genome. Glycine is the smallest among all proteinogenic amino acids, it can therefore be located in parts of the protein that are structurally forbidden to all other amino acids (e. g. tight turns). Alanine is the second smallest amino acid, it is very non-reactive and thus rarely involved directly in protein function [17]. Among deletions, glutamic acid is significantly overrepresented (*p *< 0.06). It is negatively charged and polar, and prefers to be located on the surface of proteins.

On the other hand, for insertions and deletions phenylalanine and tyrosine (both *p *< 0.003), for insertions isoleucine (*p *< 10^-4^), lysine (*p *< 0.03) and valine (*p *< 0.0006), and for deletions asparginine, leucine and tryptophan (all *p *< 0.03) are significantly underrepresented among indels. Most of these amino acids prefer to be buried within protein hydrophobic cores (phenylalanine, tyrosine, isoleucine, valine, leucine and tryptophan). Leucine is preferentially located in alpha helices, isoleucine and valine are often found in beta sheets. Asparginine and lysine predominantly reside on the surfaces of proteins [17]. Generally, all significantly underrepresented amino acids are restricted to particular positions in the protein structure. Insertions and deletions of these amino acids are likely to cause major changes in protein structure, stability and function, and are therefore strongly suppressed by purifying selection.

In order to obtain a more general survey of the underlying characteristics that dispose amino acids to be over- or underrepresented in our set, we grouped them with respect to their physio-chemical properties. Results are shown in Figure 3b. This analysis revealed that indeed small and tiny amino acids are preferentially inserted (*p *< 10^-4^) and tiny amino acids deleted (*p *< 0.05), whereas aliphatic and aromatic amino acids occur less often in inserted (*p *< 10^-6^) and hydrophobic (*p *< 0.002), aliphatic and aromatic (*p *< 10^-5^) amino acids in deleted sequence segments compared to their average abundance in protein coding regions.

Insertions and deletions in protein coding regions primarily involve amino acids that have a minor impact on the structure and function of the protein. In contrast, amino acids which are preferentially located in structurally important regions of the protein are highly suppressed. These results agree with the observed dependence of amino acid substitution rates on their local environment within the protein derived from protein alignments [10,18,19]. For example, amino acids buried in protein cores have been found to be far more conserved than those at surface positions [9].

### Structural preferences of indels

Insertion and deletion rates of amino acids depend on the structural region of the protein they are preferentially located in, as pointed out by the previous analysis. To investigate whether this effect can also directly be measured on the structural level, we retrieved secondary structure information for protein sequences affected by indels from the Protein Data Bank (PDB) [20].

**Figure 4:**
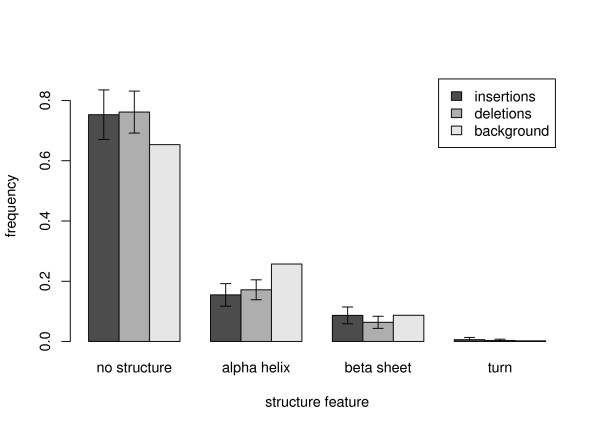
**Indel frequencies in different structural regions of proteins**. Frequency distribution of indel events in the four secondary structure categories helix, sheet, turn and no structure. The background distribution is the relative fraction of amino acids residing in each structure among all analyzed proteins. Error bars were calculated by $\Delta {ƒ}_{i}=\sqrt{N_{i}}/\sum_{j}N_{j}$, where *N*_*i *_is the total number of indels in structure *i*.

Secondary structure information could be obtained for 343 indels in our set (see methods). In Figure 4 we show the distribution of structural features (alpha helix, beta sheet, turn, no structure) among inserted and deleted coding sequence segments in comparison to the background abundance of these features in the analyzed proteins. The analysis corroborates our presumption that coding indels in human preferentially occur in protein regions lacking important secondary structure features, as has already been reported for indels derived from alignments of protein families [18] and coding indels in rodents [11]. In contrast, indels in alpha helices are significantly suppressed (*p *< 0.05). This is consistent with the fact that alpha-helices are the most robust secondary structures. For instance, they often form the skeleton of the protein. Amino acid insertions or deletions in protein regions that are supposed to form a alpha helix can have a great impact on the helical structure, since they are likely to destroy the internal periodicity of the helix. The observed suppression of indels in these regions is therefore likely to reflect the influence of purifying selection.

### Strength of selection in indel containing genes

The strong suppression of frameshifting indels, the low ratio of non-conservative indel events in our indel phase analysis, or the underrepresentation of events which presumably affect protein structure, all these findings indicate that indels in protein coding regions are exposed to a substantial amount of selective pressure. It is therefore reasonable to assume that indels preferably occur in genes where overall selection strength is lower compared to other genes.

To verify this presumption we analyzed the distribution of *dN*/*dS *ratios for indel containing genes in comparison to the background distribution of *dN*/*dS *values in all human genes. The ratio of non-synonymous substitution rate (*dN*) to synonymous substitution rate (*dS*) is a widely-used method to investigate the general strength of selection in protein coding regions. A low ratio *dN*/*dS *≪ 1 indicates strong purifying selection, while genes with *dN*/*dS *≈ 1 are usually considered to evolve under approximate selective neutrality [21]. In order to obtain meaningful estimates of *dN *and *dS *on the scale of individual genes by cross-species comparison, the divergence between the analyzed species should not be too low. *dN*/*dS *values were therefore calculated on the basis of human-mouse alignments, which could be obtained for a total of 15550 orthologous genes (see methods).

**Figure 5:**
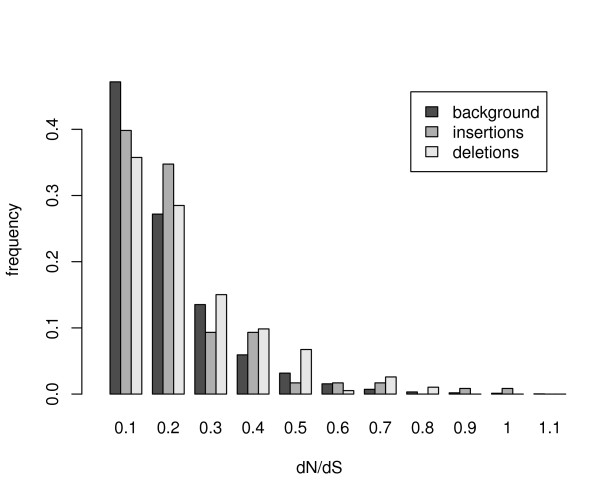
**Distribution of *dN*/*dS *values among indel containing genes**. The histograms show the measured distributions of gene frequencies with *dN*/*dS *values in binned intervals of length 0.1, starting from 0. Gene frequencies are generally peaked in the interval 0 ≤ *dN*/*dS *≤ 0.1 and decay for larger *dN*/*dS *values, indicating strong purifying selection on protein coding regions throughout evolution. However, the distributions of the subsets of genes that contain at least one insertion/deletion decay slower compared to the background distribution of all analyzed genes.

In Figure 5 we show the measured distributions of *dN*/*dS *values for all genes, compared to the subsets of genes that contain at least one coding insertion/deletion event. As expected, coding indels indeed occur preferentially in genes evolving under lower levels of selective pressure, indicated by higher *dN*/*dS *values.

We are aware of the fact that this approach determines the strength of selection on a considerably larger time-scale compared to the period where indels in our set were generated. However, the regime of strong purifying selection we are interested in, typically denotes genes associated with important biological functions. Such genes are often conserved over long evolutionary periods, and selective constraints on them are unlikely to have changed rapidly throughout recent evolution.

### Gene ontology analysis

To identify possible correlations between rates of coding indels and categories of proteins that are associated with particular molecular functions, biological processes, or cellular components, a Gene Ontology (GO) [22] analysis was performed among a broad set of 63 GO slim categories.

**Figure 6:**
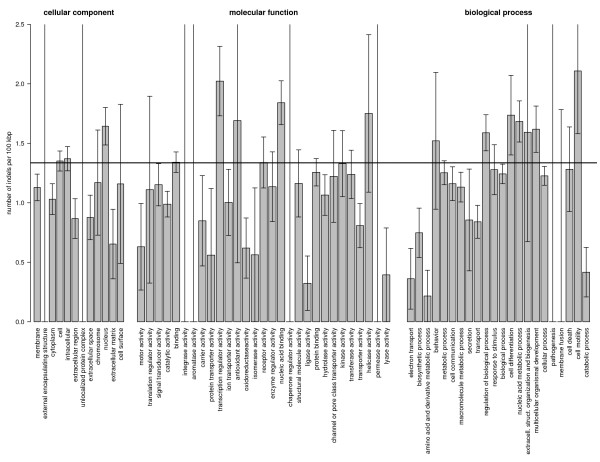
**Indel rates in 63 GO slim categories**. For each GO slim category indel rates were measured in events (insertions+deletions) per 100 kbp in the protein coding regions of all genes assigned to the particular category. The horizontal black line is the average indel rate in all protein coding regions with available GO annotation. We assumed that errors of indel rates are given by $\Delta r_{i}=\sqrt{N_{i}}/L_{i}$, where *N*_*i *_is the overall number of indels in GO slim category *i*, and *L*_*i *_is the total length of all protein coding regions assigned to that category. For GO slim categories with *N*_*i *_= 0 errors were obtained by setting *N*_*i *_= 1. The category nucleic acid metabolic process combines nucleobase, nucleoside, nucleotide and nucleic acid metabolic processes.

The standard method to investigate whether a certain GO category is over- or underrepresented in a particular subset of genes (e. g. overexpressed genes in a microarray analysis) is to compare the fraction of genes annotated by that GO category in the subset with the fraction of annotated genes in the analyzed background set. However, when analyzing indels, such an approach can be misleading if certain GO categories are systematically biased towards shorter or longer genes, since the probability of long genes to contain an indel is higher than for short genes. In order to eliminate such possible cross-correlation we directly measured the rates of coding indels in events per coding sequence length for all genes that could be mapped to our 63 GO slim categories (see methods). These rates were then compared to the average rate of coding indels in all 16,257 genes of the human genome with available GO annotation. 328 of these genes contain at least one indel of our set. The average rate of coding indels in all annotated genes was calculated to be 1 event per 75 kbp of coding sequence. All measured rates are shown in Figure 6. The most interesting result is that we found 6 categories in the ontologies molecular function and biological process which are significantly underrepresented (after applying a Bonferroni correction for multiple testing): catalytic activity (*p *< 0.04), ligase activity (*p *< 0.0003), electron transport (*p *< 0.003), amino acid and derivative metabolic process (*p *< 10^-5^), transport (*p *< 0.007), catabolic process (*p *< 0.0002). All of them are related to biochemical reactions. Suppression of indels in genes associated with these categories may be explained by the fact that biochemical reactions are very specific and are therefore highly conserved throughout evolution.

Chen et al. reported an overrepresentation of indels in genes associated with transcription regulatory activity [7]. We also measure 1.7 fold higher indel rates in this set of genes. However, we find that this overrepresentation is statistically not significant (*p *< 0.5) after correcting for multiple testing. The slight overrepresentation may actually result from the known enrichment of tandem repetitive sequences in transcription factors [23], which are therefore more prone to frequent insertions and deletions.

The category transcription regulatory activity characterizes genes that are related to the regulation of other genes. The measured higher indel rates in this class – although not significant after a conservative Bonferroni correction for multiple testing – conforms well with the hypothesis that many changes between human and chimp took not only place on the amino acid level, but also on the regulatory level [24-26]. Alongside amino acid substitutions, DNA indels in protein coding regions of regulatory genes could thereby also play an important role among the mutational processes on sequence level that drive such evolutionary changes.

In the ontology cellular component all categories besides nucleus are suppressed. Especially genes in categories related to extracellular components have lower indel rates. However, this suppression is only marginally significant (*p *< 0.06).

## Conclusion

In this study, we investigated recent DNA insertions and deletions in protein coding regions of the human genome. Mutational processes of this type were found to occur at substantially lower rates compared to indel events on a genome-wide average, indicating strong purifying selection. To enlighten particular selective constraints on coding indels in more detail, their characteristics were examined from miscellaneous angles.

DNA indels in coding sequence lead to insertions or deletions of amino acids in the translated proteins. Yet, frequencies of inserted and deleted amino acids do not resemble background amino acid frequencies in the human proteome. In particular, we found small amino acids to be preferentially inserted and deleted, while indels involving hydrophobic, aliphatic and aromatic amino acids are underrepresented. Indel rate variations could also be measured between different secondary structure regions of proteins. Amino acid insertions and deletions tend to occur in protein regions that do not form important structural domains, and are significantly underrepresented in alpha helices. We further found that indel rates in genes related to elementary biochemical reactions are subject to substantially stronger purifying selection.

Identifying selection in evolution of human proteins has drawn a considerable amount of attention since the advent of molecular sequence data. Most studies have thereby focused on the effects of amino acid substitutions [27]. With the increasing availability of single nucleotide polymorphism data, the scope has nowadays been extended to the search for events ascribed to positive selection [21,25,28]. However, one has to keep in mind that the emergence of beneficial mutants is certainly rare compared to deleterious ones. The vast majority of newly arising alleles in protein coding regions is subject to strong selective constraints. Our findings corroborate that many commonly accepted characteristics of these constraints for substitutions do also apply to amino acid insertions and deletions.

## Methods

### Identifying insertions and deletions

**Figure 7:**
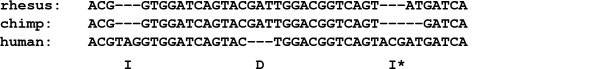
**Identifying insertion and deletion events**. The figure shows an exemplary multiple alignment of orthologous sequence segments in human, chimp and rhesus. The gap containing regions I and D can unambiguously be explained by a single insertion (I) or deletion (D) event in the human lineage since its speciation from the common ancestor with chimp. In contrast, region I* has non-overlapping gaps in chimp and rhesus and therefore requires at least two indel events. These scenarios are always ambiguous. For instance, I* can be explained by an insertion in human and a deletion in chimp, but also by a deletion in chimp and a deletion in rhesus.

Our multiple human-chimp-rhesus alignments were obtained from the Ensembl database (version 41, October 2006) [13]. They are based on the releases homo_sapiens_core_41_36c, pan_troglodytes_core_41_21 and macaca_mulatta_core_41_10a, and were generated by MLAGAN [29]. Gaps in these alignments correspond to insertion or deletion events along branches of the phylogenetic tree ((human, chimp), rhesus). We define a situation as an insertion in the human lineage since speciation from the common ancestor with chimp if the alignment has a segment of gaps in the chimp and rhesus sequences, while no gaps are present in the corresponding segment of the human sequence. Additionally, we require the gap segments in the chimp and rhesus sequences to start and end at the same position (case I in Figure 7). This is necessary since alignment regions with not exactly overlapping gap segments in chimp and rhesus cannot be explained by only one insertion event. They require at least two indel events and it is not possible to assign the events to particular branches of the phylogenetic tree in an unambiguous manner (see e. g. case I* in Figure 7). Accordingly, we define an event as a deletion in the human lineage since speciation from the common ancestor with chimp if the multiple alignment has a segment of gaps in the human sequence where no gaps are present in the chimp and rhesus sequences (case D in Figure 7). The ancestral deleted sequence is approximated by the present chimp sequence.

We applied successive filtering steps to further increase the quality of our set. The 10 bp upstream and downstream flanking regions of an identified insertion or deletion in the three species alignment were not allowed to contain more than one mismatch or gap. We also added quality constraints on the indel sequence itself. For an insertion, the number of not known nucleotides (N's) and for deletions the number of mismatches in the corresponding pairwise alignments of chimp and rhesus had to be less than 10% of the indel length.

We classified an indel to be coding if it is located within a protein coding region of an exon according to Ensembl version 41. Long insertions and deletions that are not entirely located within a preexisting exon (or insertions, which additionally insert intronic segments) were excluded from our analysis. As starting position of an indel we took the position of the first inserted nucleotide for an insertions and the position of the first changed nucleotide for a deletion.

### P-value calculations and corrections for multiple testing

P-values for significance tests were calculated using *p *= erfc(*z*/$\sqrt{2}$). The z-scores *z *measure the differences between observed values and background values in standard deviations. To correct p-values for multiple testing a Bonferroni correction was applied whenever more than one test was performed: All p-values were multiplied by the number of tests in this category.

### Simulation of indel events in coding regions

Two test sequences were generated, one by concatenating the protein coding nucleotide sequences of all genes containing an insertion from our set, the other by concatenating the corresponding sequences for all deletions from our set. Insertion events were simulated on the first test sequence, deletions on the second test sequence, according to the following procedure:

1. The length *l *of the insertion (deletion) was drawn from the length distribution of insertions (deletions) in our set (Figure 1). For simplicity, indel length was restricted to 21 bps.

2. According to the genome-wide frequency of tandem duplication insertions (deletions from tandem duplicates) among all insertions (deletions) for the particular indel length *l *(numbers taken from [8]), it was chosen whether the insertion (deletion) should be a tandem duplication (deletion from a preexisting tandem duplicate), or not.

3. A random position *p *in the test sequence was selected.

4. In case of a tandem duplication insertion, the sequence segment of length *l *starting at position *p *in the test sequence was duplicated and inserted again at position *p*. In case of an insertion that was not chosen to generate a tandem duplication, a sequence segment of length *l *was generated by independently drawing each nucleotide from the distribution of base frequencies in the test sequence. The randomly generated segment was then inserted at position *p*. In case of a deletion it was checked whether deleting the *l *nucleotide long sequence segment starting at position *p *would effectively constitute a deletion of one copy of a preexisting tandem duplicate, or not. If the result coincided with the scenario chosen for the particular deletion, the segment was deleted. Otherwise the procedure was reiterated from 3 until a suitable position *p *was found.

5. It was checked whether the resulting insertion (deletion) event was conservative or not.

Succeeding simulation runs were always performed on the original test sequences meaning that the generated indel of a previous run was not incorporated in the test sequence for the next run. The frequency of non-conservative events were calculated on the basis of 10^6 ^simulation runs for insertions and the same number of runs for deletions.

### Inserted and deleted amino acids

Amino acid sequences of insertions were derived by translating all codons that overlap with the inserted DNA segments. In case of deletions, the deleted segments were re-inserted in the human sequence and all overlapping codons were translated. Notice that by this procedure we also take into account only partially affected amino acids at the boundaries of phase 1 and 2 indels. Frequencies of the different amino acids were obtained by counting their occurrences in the inserted/deleted amino acid sequences, divided by the overall number of all amino acids in inserted/deleted sequences.

We assigned amino acids to 10 overlapping groups according to their physio-chemical properties: hydrophobic, aliphatic, aromatic, small, tiny, proline, polar, charged, positive and negative [30]. The frequency of inserted/deleted amino acids in each group was calculated by summing up the number of all inserted/deleted amino acids assigned to that group, divided by the overall number of all inserted/deleted amino acids.

For the background model, we measured the frequencies of amino acids in all protein coding regions of the human genome which are annotated by Ensembl. P-values for amino acid distributions were multiplied by a factor 20 and for the group distribution by a factor 10 to correct for multiple testing.

### Retrieving protein secondary structure

For each indel in our set the sequence of its encompassing protein was blasted against the PDB using blastp from the NCBI QBlast system with default parameters to obtain information on the secondary structure of the protein. In case of a deletion, we blasted the reconstructed ancestral sequence. If more than one hit was reported from the PDB, we chose the first found PDB id which overlaps with the whole indel. The PDB assigns the structural features helix, sheet, turn, or no structure to every amino acid position of the protein. For each of the four structural features we counted the number of indels in our set that reside in a protein region annotated by the structure. If an indel covers more than one structural feature, we weighted each feature by the relative fraction of the length it covers of the indel. For example, a 9 bp long indel where the first 3 bp reside in a protein region annotated as turn, while the last 6 bp are annotated as no structure, adds 1/3 to feature "turn" and 2/3 to feature "no structure". The obtained counts for each structural feature were then divided by the number of all inserted/deleted amino acids with available structural annotation.

For the background model, we added for each structural feature the number of amino acids annotated with the feature in all analyzed PDB sequence segments that overlap with the blasted protein sequence, and divided it by the overall length of these segments. As 4 structural categories were investigated all p-values were multiplied by a factor 4 to correct for multiple testing.

### Estimating *dN*/*dS *values from human-mouse alignments

Amino acid sequences and the corresponding nucleotide alignments for orthologous genes in human and mouse were retrieved from Ensembl. Values for *dS *and *dN *in these alignments were computed with codeml (from PAML, v3.15 [31]) assuming the F3×4 codon frequency model.

### Measuring indel rates in GO slim categories

All human genes in Ensembl with available GO annotation were mapped to their corresponding GO slim categories using goaslim.map [32]. Notice that a gene might be attributed to several GO slim categories. For all genes in a particular GO slim category we then counted all coding indels in our set which are located within these genes, and divided this number by the total length of all protein coding regions in the category. This way we retrieved category-specific indel rates in events per bp. For the average rate we counted the number of coding indels in all genes that are annotated with GO terms and divided this number by the total length of coding regions in all annotated genes.

The main Gene ontologies cellular component, molecular function, and biological process are independent from each other. Within one group p-values were multiplied by factors 12, 29, and 22, in order, to correct for multiple testing.

## Authors' contributions

NC carried out the data analysis and wrote the first version of the manuscript. PM critically revised the manuscript. PM contributed to the design of the study, which was coordinated by PA. All authors read and approved the final manuscript.
